# mGluR5 Mediates Dihydrotestosterone-Induced Nucleus Accumbens Structural Plasticity, but Not Conditioned Reward

**DOI:** 10.3389/fnins.2018.00855

**Published:** 2018-11-20

**Authors:** Kellie S. Gross, Kelsey M. Moore, Robert L. Meisel, Paul G. Mermelstein

**Affiliations:** ^1^Graduate Program in Neuroscience, University of Minnesota, Minneapolis, MN, United States; ^2^Department of Neuroscience, University of Minnesota, Minneapolis, MN, United States

**Keywords:** nucleus accumbens, dihydrotestosterone, dendritic spine, conditioned place preference, mGluR5

## Abstract

Gonadal hormones play a vital role in driving motivated behavior. They not only modulate responses to naturally rewarding stimuli, but also influence responses to drugs of abuse. A commonality between gonadal hormones and drugs of abuse is that they both impact the neurocircuitry of reward, including the regulation of structural plasticity in the nucleus accumbens (NAc). Previous hormonal studies have focused on the mechanisms and behavioral correlates of estradiol-induced dendritic spine changes in the female NAc. Here we sought to determine the effects of androgens on medium spiny neuron (MSN) spine plasticity in the male NAc. Following treatment with the androgen receptor agonist dihydrotestosterone (DHT), MSNs in castrated male rats exhibited a significant decrease in dendritic spine density. This effect was isolated to the shell subregion of the NAc. The effect of DHT was dependent on mGluR5 activity, and local mGluR5 activation and subsequent endocannabinoid signaling produce an analogous NAc shell spine decrease. Somewhat surprisingly, DHT-induced conditioned place preference remained intact following systemic inhibition of mGluR5. These findings indicate that androgens can utilize mGluR signaling, similar to estrogens, to mediate changes in NAc dendritic structure. In addition, there are notable differences in the direction of spine changes, and site specificity of estrogen and androgen action, suggesting sex differences in the hormonal regulation of motivated behaviors.

## Introduction

Gonadal hormones are critical modulators of motivated behavior. Estrogens, progestins, and androgens control the expression of a range of naturally motivated behaviors (Beatty, 1979; Fahrbach and Pfaff, 1982; Pfaff, 1982), as well as influencing the acquisition, escalation, and relapse behaviors associated with addiction (Anker and Carroll, 2011; Becker et al., 2012). Therefore, identifying the neurobiological mechanisms by which hormones influence the reward circuitry of the brain is key to understanding their underlying impact on motivated behaviors, and to suitably tailor treatments in both men and women suffering from addiction (Becker et al., 2012).

One way that hormones can influence brain regions associated with reward is through modulating structural plasticity. In the nucleus accumbens (NAc), a primary component of the mesolimbic dopamine system, changes in dendritic spine density and morphology of medium spiny neurons (MSNs) occur in response to both natural rewards and drugs of abuse (Pitchers et al., 2010; Russo et al., 2010; Staffend and Meisel, 2012; Staffend et al., 2014). The structural changes induced by drugs of abuse in this region are incredibly persistent and have been found to correlate with aspects of drug-mediated behavior (Li et al., 2004; Robinson and Kolb, 2004), suggesting that they may be functionally relevant to long-lasting changes in synaptic signaling and behavior. Recent evidence has shown that sex steroid hormones also modulate spine density in the NAc. Our own lab has found that estradiol decreases MSN spine density in the NAc core (NAcC) of ovariectomized female rodents (Staffend et al., 2011; Peterson et al., 2014). Other work has shown that high doses of testosterone given to intact male rats in a model of anabolic-androgenic steroid abuse decreased spine density of MSNs, though this was found in the NAc shell (NAcSh) (Wallin-Miller et al., 2016).

These findings provide initial evidence that sex steroid hormones may differentially affect spine plasticity between the two subregions of the NAc. However, further study of androgen action in males is required to determine if this phenomenon exists outside of models of steroid abuse. These data also show a striking similarity in that both estrogens and androgens decrease spine density in the NAc, an unusual effect considering that both classes of hormone typically increase the density of spines in other regions of the brain (Woolley and McEwen, 1992; Leranth et al., 2003; Hajszan et al., 2007; de Castilhos et al., 2008; Christensen et al., 2011; Khan et al., 2013). Previous work in our lab has developed a model of the molecular mechanisms underlying estradiol-induced spine decreases in the female NAcC, finding that these changes occur through activation of mGluR5 and endocannabinoid signaling (Peterson et al., 2014, 2016). This mGluR5 signaling mechanism was also found to be critical for estradiol-driven potentiation of psychostimulant-induced behaviors in female rodents (Martinez et al., 2014, 2016). Whether hormone-induced NAc spine decreases and behavioral effects in males are also driven by mGluR5 is unknown. As mGluR5 activity has previously been associated with behavioral responses to drugs of abuse, and is considered a potential therapeutic target for addiction (Olive, 2009; Cleva and Olive, 2012), it is particularly important to understand how sex steroid hormones may work with this receptor to influence mesolimbic regions. Therefore, the aims of this study were to explore the effects and mechanism of androgen action on spine plasticity in the male NAc, with specific interest on the action of mGluR5.

## Materials and Methods

### Animals

Castrated Sprague-Dawley rats (200–225g, 8–9 weeks old) were purchased from Envigo Laboratories (Indianapolis, IN, United States). Animals were pair housed and kept on a 12:12 light–dark cycle (lights out at 10 a.m.) with food and water *ad libitum*. Animals were allowed to habituate to the research facility for 5 days prior to the start of any experiment. All animal procedures were in accordance with the National Institutes of Health Guidelines for the Care and Use of Laboratory Animals and were approved by the Animal Care and Use Committee at the University of Minnesota.

### Drugs and Hormones

5α-androstan-17β-ol-3-one (DHT; Steraloids Inc.; Newport, RI, United States) was dissolved in cottonseed oil, and 1.5 mg was injected subcutaneously (s.c.) in a volume of 0.2 ml. All other drugs were obtained from Tocris Bioscience (Minneapolis, MN, United States). The ERβ agonist diarylpropionitrile (DPN) was dissolved in cottonseed oil at a concentration of 1 mg/ml and injected s.c. at dose of 1 mg/kg. 2-methyl-6-(phenylethynyl)pyridine hydrochloride (MPEP) was dissolved in physiological saline on the day of injection at a concentration of 1 mg/ml and was injected intraperitoneally (i.p.) 30 min prior to DHT administration at a dose of 1 mg/kg. This dose of MPEP has previously been shown to block estradiol-induced structural and behavioral effects (Martinez et al., 2014; Peterson et al., 2014). For site-specific mGluR5 activation, (RS)-2-chloro-5-hydroxyphenylglycine sodium salt (CHPG) was dissolved in sterile saline at a concentration of 20 mg/ml and infused into the NAc at a dose of 10 μg/0.5 μl/side. Infusion of an equal volume of saline (0.5 μl) was used for a vehicle control. The CB1 receptor inverse agonist, N-(piperidin-1-yl)-5-(4-iodophenyl)-1-(2,4-dichlorophenyl)-4-methyl-1H-pyrazole-3-carboxamide (AM251) was dissolved in DMSO to a concentration of 100 mM, aliquoted and stored at -20°C. On the day of injections, aliquots were thawed and diluted in 0.5% methylcellulose (Sigma-Aldrich, St. Louis, MO, United States) to a working concentration of 1 mg/ml. AM251 or a corresponding vehicle solution was injected i.p. at a dose of 1 mg/kg approximately 30 min prior to the start of the intracranial infusions. This dose of AM251 has previously been shown to block estradiol-mediated dendritic spine changes (Peterson et al., 2016). Animals were sacrificed 24 h after hormone or CHPG treatment and tissue was then processed for dendritic spine analysis.

### Surgical Microinjections

Twenty minutes prior to surgery, animals were given an s.c. injection of 5 mg/ml/kg carprofen (Zoetis, Parsippany, NJ, United States) to induce analgesia. Animals were also given an s.c. injection of 10 mg/ml/kg Baytril (Bayer DVM, Shawnee Mission, KS, United States) at the time of surgery to prevent infection. Animals were anesthetized with a mixture of 2.5–4% isoflurane (Piramal Critical Care, Bethlehem, PA, United States)/oxygen and placed in a stereotaxic apparatus. CHPG or saline was injected bilaterally via a Hamilton microinjection syringe at coordinates targeting the NAcC-shell border: AP: +1.80 mm from bregma, ML: +-1.50 mm from bregma, DV: -6.20 mm from dura. Volumes of 0.5 μl, designed to affect both the core and shell regions, were infused manually over the course of 2.5 min. The injection needle was then left in place for an additional 2.5 min to allow for drug diffusion. Following surgery, animals were monitored until they recovered ambulatory posture and were then observed for eating and drinking behavior and any signs of discomfort before returning them to the colony. Post-operative analgesia and antibiotics were not required as all animals were sacrificed within 24 h of the time of surgery.

### Tissue Preparation

Tissue was prepared and ballistically stained with DiI following previously established protocols (Staffend and Meisel, 2011). Twenty-four hours after drug or hormone treatment, animals were given an overdose of Beuthanasia-D (390 mg/ml sodium pentobarbital, 0.35 ml i.p., Schering, Union, NJ, United States), injected with 0.25 ml heparin into the left ventricle of the heart and then transcardially perfused with 25 mM phosphate buffered saline (PBS, pH = 7.2) for 3 min followed by 1.5% paraformaldehyde in 25 mM PBS for 20 min. Brains were removed, coronally blocked, and then post-fixed in 1.5% paraformaldehyde and PBS for 1 h before being stored in 25 mM PBS. A Leica VT1000 S Vibratome (Buffalo Grove, IL, United States) was used to coronally slice brains into 200–300 μm thick sections through the striatum. Sections were kept in 25 mM PBS until the time of DiI labeling.

### DiI Labeling

DiI “bullets” were made using Tefzel tubing (Bio-Rad, Hercules, CA, United States) pretreated with 15 mg/ml polyvinylpyrrolidone (PVP). Two milligrams of DiI (Molecular Probes, Carlsbad, CA, United States) was dissolved in 100 μl dichloromethane and applied to 90 mg of 1.3 μm tungsten particles (Bio-Rad). Particles were suspended in 10 ml of PVP solution and then sonicated for 13 min with intermittent vortexing. The suspension was quickly pulled through the prepared tubing and allowed to settle before the remaining PVP was expelled. Nitrogen gas flow was used to dry the tubing for 20 min, and then the tubing was cut into 1.33 cm bullets. DiI-coated tungsten particles were delivered to the tissue using a Helios Gene Gun (Bio-Rad) with a modified barrel, 40 mm spacer, and 70 μm mesh filter. After loading bullets into the gun, PBS was removed from wells containing brain sections, and one bullet was shot per section using helium gas expulsion at 100 PSI. Sections were the stored overnight in the dark in 25 mM PBS and post-fixed the following morning in 4% paraformaldehyde in PBS for 1 h. Sections were mounted on slides and coverslipped with FluorGlo mounting media for lipophilic dyes (Spectra Services, Ontario, NY, United States).

### Confocal Imaging

**FIGURE 1:**
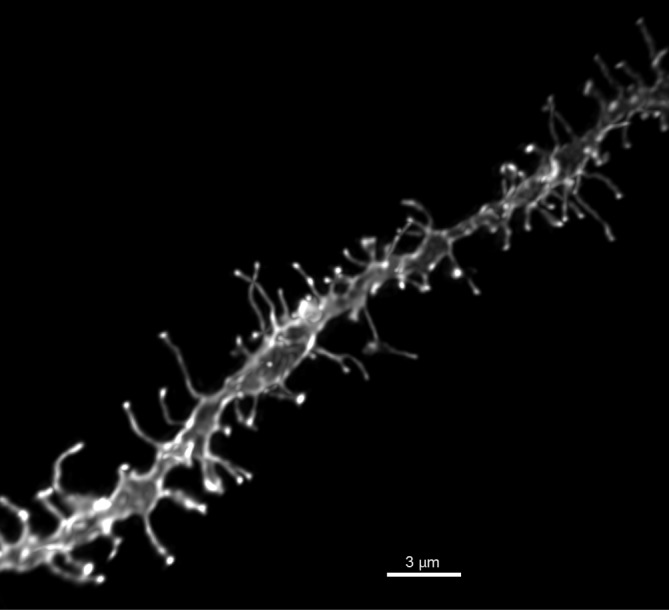
Representative image of DiI dendritic segment. High power (60×) reconstruction of a DiI labeled NAcSh MSN dendritic segment, scale bar 3 μm.

Dendritic segments (Figure 1) were imaged on a Leica TCS SPE confocal microscope using a Leica PLAN APO 63×, 1.4 NA oil immersion objective (11506187, Leica, Mannheim, Germany) and Type LDF immersion oil (Cargille, Cedar Grove, NJ, United States). Whole cells were imaged using a 20× air objective. All images were taken at an *xy* pixel distribution of 512 × 512 and a frequency of 400 Hz. Whole cell images taken at step size 1 μm in the z-axis were reconstructed in the Leica LAS AF software to measure the distance from the soma to each dendritic segment. For each neuron, images of 2–3 dendritic segments (70–200 μm from soma) were taken at a step size of 0.12 μm and optical zoom of 5.6, with the laser power and photomultiplier being adjusted to capture the dendrite in its full dynamic range. Images from 2 to 3 MSNs in both the NAcC and NAcSh were taken. For each animal, a minimum of six dendritic segments from a brain region was required to be included in the analysis. Data from 4 to 8 animals were collected for each treatment group in an experiment (see figure legends for specific group sample sizes). Prior to analysis, images were processed through 3D deconvolution using AutoQuant X3 AutoDeblur software (Media Cybernetics, Bethesda, MD, United States).

### Quantitation

All quantitation was completed by an experimenter blinded to treatment condition. Z-stacks were reconstructed in the Surpass module of Imaris software (Bitplane Inc., St. Paul, MN, United States), and then dendrites were manually traced using the Filament tool and Autodepth function. A 3D reconstruction of the dendritic shaft and spines was rendered using the diameter function with a contrast threshold of 0.7, and data on spine density, spine length, and head diameter was collected for each segment. Spine densities for each segment (collected as average spine density/10 μm) were averaged across each cell and then within each brain region for each animal, providing a region-specific spine density average for each animal that was then used for statistical analysis. Measurements of spine length and head diameter were pooled for each treatment condition then plotted as binned cumulative probability distributions (bin sizes: spine length, 0.5 μm; head diameter, 0.1 μm).

### Conditioned Place Preference (CPP)

The conditioned place preference (CPP) apparatus consisted of two lateral chambers (60 × 45 × 38 cm) connected by a clear central chamber (37 × 22 × 38 cm). The lateral chambers were differentiated by the color of the walls and the type of bedding on the chamber floor: one lateral chamber was gray and contained Aspen bedding (Harlan Laboratories, IN, United States) while the other lateral chamber was white and contained 1/8” corncob bedding (Harlan Laboratories).

Conditioned place preference for DHT was based on prior publications (Alexander et al., 1994; Schroeder and Packard, 2000). Conditioning took place over a total of 10 days, 1 h after lights out, under red light conditions. To establish an initial preference for one of the lateral compartments, the first day consisted of a filmed pretest where animals were allowed to freely explore the entire apparatus in a drug-free state for a total of 10 min. All animals preferred the compartment with gray walls and Aspen bedding. Following established procedures designed to detect changes in preference when using a biased apparatus (Tzschentke, 2007), DHT injections (1.5 mg/0.2 ml in cyclodextrin, i.p., Steraloids) were given just prior to the rat’s placement for 30 min in the initially non-preferred corncob/white side, whereas cyclodextrin vehicle injections (0.2 ml 45% 2-hydroxypropyl-β-cyclodextrin, i.p., Abcam) were given prior to placement for 30 min in the initially preferred Aspen/gray side over the course of conditioning. All animals received eight alternating days of conditioning (4 DHT days and 4 saline days), with animals cross-balanced on each day. To test the hypothesis that mGluR5 mediates the rewarding effects of DHT, half of the animals received saline vehicle injections 30 min prior to placement in the chamber paired with DHT, while the other half were given MPEP injections (1 mg/kg in saline, i.p.) 30 min prior to placement in the chamber paired with DHT. As a control for injection stress, all animals also received saline vehicle injections 30 min prior to placement in the cyclodextrin-paired chamber. Following the eight training days, animals were filmed in a 10-min final preference posttest where they were allowed access to all three compartments to assess their final preference. Time spent in the non-preferred (DHT paired) compartment was then compared between pretest and posttest for both groups.

### Data Analysis

All data analysis was conducted in SPSS software. For spine density, groups were compared using either a Student’s *t*-test or a two-way ANOVA. Significant interactions were followed up using a test of simple main effects. The DiI method on occasion can introduce variability in the efficiency of labeling due to non-biological factors (Staffend and Meisel, 2011). Consequently, we remove extreme statistical outliers from the morphological analyses. Data were examined for these extreme outliers (more than three box-lengths from the edge of the box in a boxplot) with the measurements from an individual animal removed. Only three outliers were removed across all experiments. Morphology distributions were compared to the control group using a two-sample Kolmogorov–Smirnov test. For CPP, our goal was to determine whether individual treatment conditions had rewarding consequences. To maximize statistical power pretest and posttest times in the non-preferred compartment for each group were compared using paired sample *t*-tests. Data are presented as mean ± SEM unless otherwise noted. For all tests, results were considered significant if *p* < 0.05.

## Results

**FIGURE 2:**
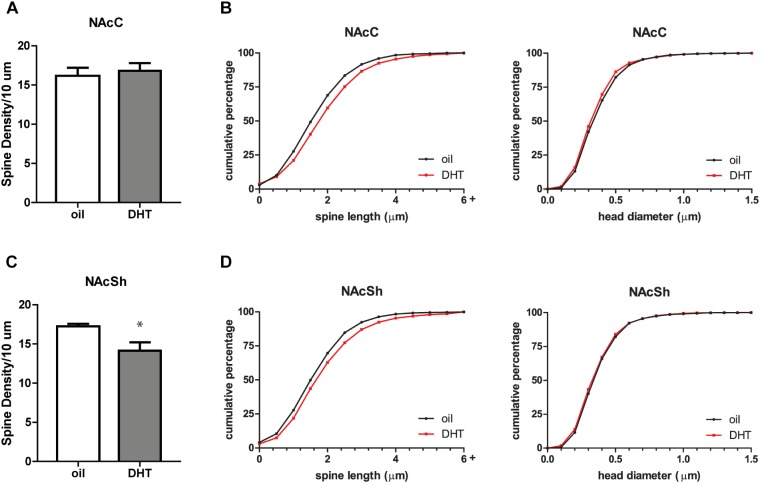
DHT decreases spine density in the NAcSh of castrated male rats. **(A)** Average spine density per 10 μm of MSNs in the NAcC 24 h after systemic administration of DHT (1.5 mg) or oil control. **(B)** Spine morphology of MSNs in the NAcC as assessed by binned cumulative probability distributions of spine length and head diameter in oil and DHT treated groups. **(C)** Average spine density per 10 μm of MSNs in the NAcSh 24 h after systemic administration of DHT (1.5 mg) or oil control. **(D)** Spine morphology of MSNs in the NAcSh in oil and DHT treated groups. *n* = 6 animals per group with spine density averaged from 6 to 9 dendritic segments per each subregion for each animal, ^∗^*p* < 0.05.

**FIGURE 3:**
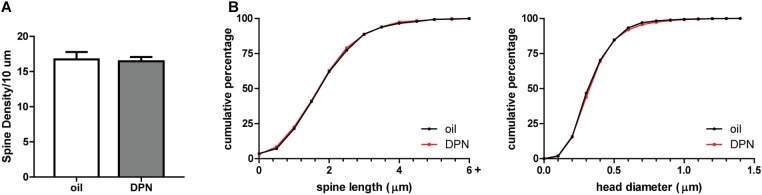
ERβ activation does not influence spine plasticity in the NAcSh of castrated male rats. **(A)** Average spine density per 10 μm of MSNs in the NAcSh 24 h after systemic administration of DPN (1 mg/kg). **(B)** Spine morphology of MSNs in the NAcSh as assessed by binned cumulative probability distributions of spine length and head diameter in vehicle and DPN treated groups. *n* = 4 animals per group with spine density averaged from 6 to 9 dendritic segments per animal.

To determine if androgen activity affects MSN spine density in the NAc, we treated castrated male rats with DHT, a potent agonist of the androgen receptor (AR). Spine density, head diameter, and length were measured from confocal images of dendritic segments of NAcC and NAcSh MSNs (Figure 1) 24 h after administration of DHT or oil control, a time point at which hormone-mediated spine changes often peak (Woolley, 1998). Systemic DHT treatment had no effect on spine density in the NAcC (Figure 2A), but did significantly reduce spine density in the NAcSh [Figure 2C, *t*(9) = 2.53, *p* = 0.032]. DHT had no effect on either spine length or head diameter in either subregion (Figures 2B,D). Although DHT cannot be aromatized into estradiol, a metabolite of DHT, 3β-androstanediol, is active at estrogen receptor β (ERβ) (Pak et al., 2005). Thus, to determine whether ERβ activation mediates DHT-induced spine plasticity in the male NAcSh, the ERβ agonist DPN was systemically administered to castrated males, and spine density and spine morphology were measured 24 h later. Systemic DPN had no effect on NAcSh spine density or morphology (Figure 3), suggesting that observed DHT effects are mediated by activation of ARs.

**FIGURE 4:**
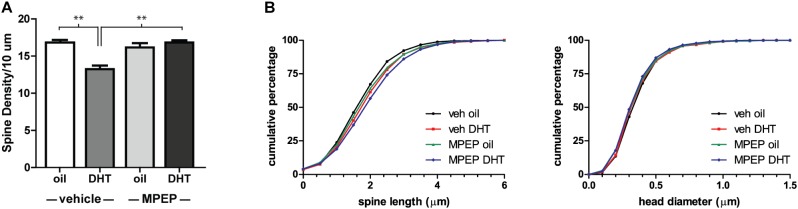
DHT-induced spine plasticity is dependent on mGluR5. Spine density and morphology were measured in castrated male rats receiving veh/MPEP (1 mg/kg) 30 min prior to injection of oil/DHT (1.5 mg). **(A)** Average spine density per 10 μm of MSNs in the NAcSh 24 h after hormone treatment. **(B)** Spine morphology of MSNs in the NAcSh 24 h after hormone treatment as assessed by binned cumulative probability distributions of spine length and head diameter. *n* = 4–5 animals per group with spine density averaged from 6 to 9 dendritic segments per animal, ^∗∗^*p* < 0.001.

This spine density decrease in the NAcSh is reminiscent of NAcC spine decreases observed in female animals 24 h following the administration of estradiol. Further study of the mechanism of this estradiol-induced effect has shown that it is dependent on the activation of mGluR5 (Peterson et al., 2014). To determine if mGluR5 signaling is required for the DHT-induced spine decrease in males, we looked at the effect of DHT treatment on NAcSh spine density in animals pretreated with the mGluR5 antagonist, MPEP, or vehicle control. We found that the effect of DHT was dependent on mGluR5 activation [Figure 4A, hormone by drug interaction: *F*(1,14) = 21.97, *p* < 0.001], with animals that received MPEP prior to DHT exhibiting a higher spine density than those that were vehicle treated [*F*(1,14) = 25.69, *p* < 0.001]. Again, this plasticity appears to be restricted to changes in spine density, as there was no influence of treatment condition on spine length or head diameter (Figure 4B).

**FIGURE 5:**
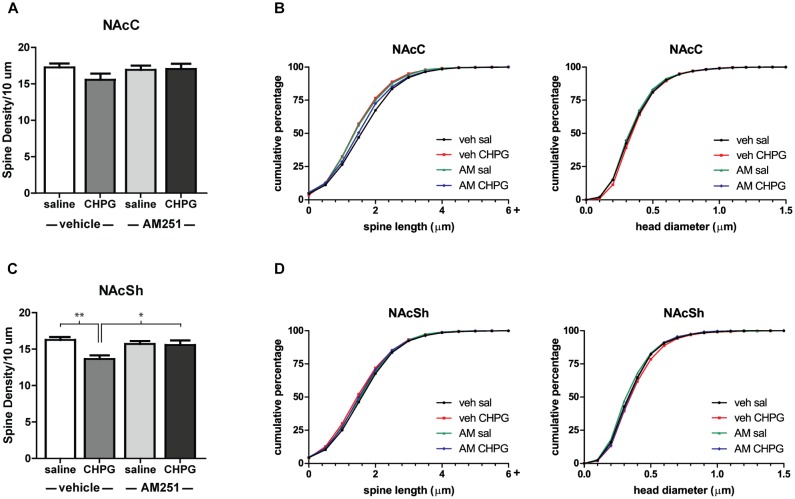
Local mGluR5 activation decreases NAcSh spine density and is endocannabinoid dependent. Spine density and morphology were measured in male rats receiving systemic veh/AM251 (1 mg/kg) 30 min prior to local injection of veh/CHPG (10 μg/side) into the NAc. **(A)** Average spine density per 10 μm of MSNs in the NAcC 24 h after drug infusion. **(B)** Spine morphology of MSNs in the NAcC 24 h after drug infusion as assessed by binned cumulative probability distributions of spine length and head diameter. **(C)** Average spine density per 10 μm of MSNs in the NAcSh 24 h after drug infusion. **(D)** Spine morphology of MSNs in the NAcSh 24 h after drug infusion. *n* = 7–8 animals per group with spine density averaged from 6 to 9 dendritic segments per each subregion for each animal, ^∗^*p* < 0.05, ^∗∗^*p* < 0.001.

The restriction of DHT-induced spine changes to the NAcSh suggests an interesting sex difference in how primary gonadal hormones and mGluR5 influence NAc structure. In female rodents, estradiol/mGluR5 interactions only drive spine decreases in the NAcC (Peterson et al., 2014), despite the ability of both systemic and local mGluR5 activation to decrease spine density throughout the NAc (Gross et al., 2016). To see if this sex difference extends to mGluR5 signaling independent of hormonal manipulation, the mGluR5 agonist, CHPG, was infused into the NAc of intact males, and spines were analyzed 24 h later. Compared to infusion of saline, CHPG reduced spine density in the NAcSh [Figure 5C, main effect of CHPG: *F*(1,26) = 14.09, *p* = 0.010] and had no effect on spines in the NAcC (Figure 5A). Thus, activation of mGluR5 throughout the NAc produces the same subregion-specific spine changes as systemic DHT. In addition to assessing the effect of CHPG in this region, the role of endocannabinoid signaling in this process was tested. Activation of mGluR5 in the NAc is known to lead to the synthesis and release of endocannabinoids (Alger and Kim, 2011), and this mechanism has previously been implicated in estradiol-induced spine changes in females (Peterson et al., 2016). Endocannabinoid signaling also appears to mediate mGluR5 effects on spine plasticity in the NAcSh of male rats, as systemic pretreatment with the CB1R inverse agonist AM251 influenced the effect of CHPG on spines [drug by drug interaction: *F*(1,26) = 11.38, *p* = 0.019]. Specifically, AM251 pretreatment abolished the spine density decreases in the NAcSh following intra-accumbal CHPG [Figure 5C, *F*(1,26) = 7.50, *p* = 0.011]. Neither CHPG nor AM251 influenced head diameter or spine length in either region (Figures 5B,D).

**FIGURE 6:**
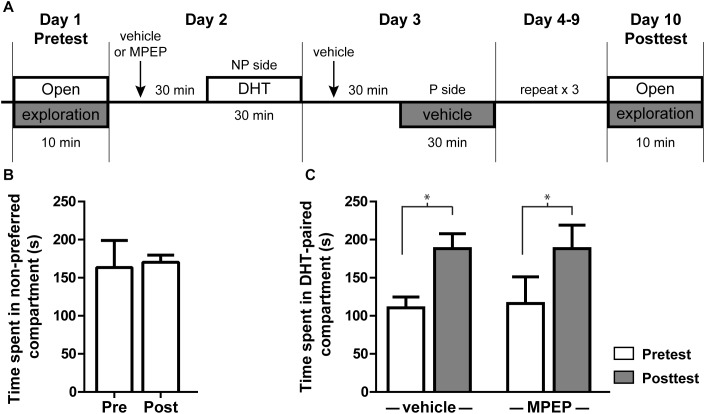
DHT conditioned place preference is not dependent on mGluR5. **(A)** Schematic of conditioned place preference behavior. Initial preference was determined with a 10 min open exploration pretest on Day 1. On the following 8 days animals were given an i.p. injection of either vehicle or MPEP, then 30 min later were given an i.p. injection of DHT and contained on the non-preferred side (NP) or vehicle and contained on the preferred side (P). On day 10, animals were allowed to freely explore the apparatus for 10 min during the posttest period. **(B)** Time spent on the non-preferred compartment during the pretest and posttest for animals injected with vehicle 30 min prior to conditioning and vehicle on both sides of the conditioning apparatus. **(C)** Effect of pretreatment with veh/MPEP on time spent in the DHT-paired compartment. *n* = 7–8 animals per group, ^∗^*p* < 0.05.

Administration of exogenous androgens is known to be rewarding and produces CPP (Alexander et al., 1994). Infusion of testosterone or its metabolites specifically into the NAcSh is sufficient to produce this effect (Frye et al., 2002), suggesting one behavioral outcome of androgen-induced plasticity in this region. To determine if mGluR5 signaling is required for androgen-induced CPP, we looked at the effect of blocking mGluR5 activity prior to DHT injection. In this CPP model, animals were given i.p. injections of either vehicle or MPEP 30 min prior to conditioning with DHT (Figure 6A). All animals initially preferred the Aspen/gray compartment over the corncob/white, a preference that remains stable in animals injected with saline on both sides of the conditioning apparatus during the behavioral procedure (Figure 6B). Regardless of MPEP pretreatment, the amount of time spent in the non-preferred compartment during the 10 min posttest period was significantly increased after conditioning with DHT [Figure 6C; *t*(7) = 3.027, *p* = 0.019 for saline group; *t*(6) = 2.627, *p* = 0.039 for MPEP group], while the amount of time spent in the initially preferred (vehicle paired) compartment was decreased [*t*(7) = 5.776, *p* < 0.001 for the saline group; *t*(6) = 5.206, *p* < 0.002 for the MPEP group]. These data suggest that systemic blockage of mGluR5 signaling was not sufficient to influence the acquisition of DHT-induced CPP.

## Discussion

Although preliminary evidence suggested that androgens can modulate spine density in the NAc (Wallin-Miller et al., 2016), this effect had yet to be thoroughly characterized and the molecular mechanisms and behavioral outcomes of this plasticity were unknown. Here we used DHT, a potent agonist of ARs, in castrated rats to find evidence that AR activation selectively decreases MSN spine density in the NAcSh. The restriction of spine decreases to the NAcSh seen here is in line with previously observed testosterone-induced spine decreases in a rat model of anabolic steroid abuse, suggesting that this subregion is a specific target for androgen-induced structural remodeling (Wallin-Miller et al., 2016). Furthermore, we provide evidence that this occurs through a novel mechanism: DHT activation of mGluR5 signaling. These findings have implications both for the role of androgen signaling in motivated behavior and for the mechanisms of androgen action throughout the brain.

Steroid hormone regulation of mGluR signaling has thus far been focused on the study of estradiol (Micevych and Mermelstein, 2008). In multiple brain regions it has been found that surface localized estrogen receptors ERα and ERβ couple to and regulate the activity of various mGluR subtypes, exerting effects on structural plasticity, synaptic physiology, and behavior (Micevych and Mermelstein, 2008; Christensen et al., 2011; Boulware et al., 2013; Martinez et al., 2014, 2016; Peterson et al., 2014). Palmitoylation and interaction with caveolin are also molecular requirements for the coupling of ERs to mGluRs (Boulware et al., 2007; Meitzen et al., 2013). There is reason to think that this signaling model might generalize to other steroid hormones. ARs are also shown to localize to the cell membrane, a process that, similar to ERs, is dependent on the post-translational modification S-palmitoylation and interaction with integral membrane caveolin proteins (Tabori et al., 2005; Pedram et al., 2007; Bennett et al., 2009). Use of DHT, which cannot be aromatized into estradiol, and demonstration that ERβ activation, which can occur via DHT metabolites, has no effect on spine plasticity in the male NAcSh suggests that the observed effects are AR dependent. Additionally, the ability of local microinjection of CHPG into the NAc to produce the same structural effects of systemic DHT suggests that DHT activation of mGluR5 may be occurring site-specifically. The fact these local mGluR5-induced spine changes are mediated by endocannabinoid signaling further supports a common mechanism for both estradiol and DHT-induced spine changes (Peterson et al., 2016). We are keenly interested in the exact nature of this putative AR/mGluR5 interaction in the NAcSh and to determine if other androgen-mediated effects depend on mGluR signaling.

Our findings that both DHT and mGluR5 mediated spine plasticity are limited to the NAcSh uncovers interesting sex differences in both NAc mGluR5 signaling and its regulation by primary gonadal hormones. In females, mGluR5 activation decreases spine density in both the NAcC and NAcSh (Gross et al., 2016). The restriction of mGluR5-induced spine decreases to the NAcSh in males, therefore, indicates intrinsic organizational differences regarding how mGluR5 signaling influences this region between the sexes. This is in line with broader evidence that both sex differences in hormonal activation and intrinsic organization exist in the reward circuitry of the brain, with differences favoring greater responsivity in females to drugs of abuse (Hu et al., 2003; Becker and Hu, 2008). In the case of mGluR5 structural plasticity, estradiol seems to take advantage of the intrinsic sex difference, driving mGluR5 spine decreases only in the NAcC (Peterson et al., 2014). These structural changes are likely to be functionally relevant as dendritic spine changes specifically in the NAcC are correlated with the expression of psychostimulant-induced locomotor sensitization and in females, estradiol-enhancement of psychostimulant sensitization depends on the activation of mGluR5 (Li et al., 2004; Martinez et al., 2014). Conversely, the lack of androgen/mGluR5 activity in the NAcC may explain the more minimal effect of circulating gonadal hormones on psychostimulant-induced behaviors in males (Hu et al., 2003; Jackson et al., 2005).

In both males and females it appears that the endocannabinoid system is an important mediator of spine plasticity in the NAc. Carvalho et al. (2016) show that repeated agonism of cannabinoid receptors decreases MSN spine density in the male NAc. In females, spine decreases in the NAcC following repeated administration of estradiol and cocaine are dependent on endocannabinoid signaling (Peterson et al., 2016). Here we observe that endocannabinoid signaling following a single infusion of mGluR5 agonist is necessary for structural plasticity in the male NAcSh. Synthesis and release of endocannabinoids occur following activation of Gq coupled GPCRs, such as mGluR5. In the NAc, endocannabinoid signaling is critical for the induction of mGluR5-dependent LTD at corticostriatal synapses via reduction of presynaptic glutamate release (Robbe et al., 2002; Jung et al., 2005; Alger and Kim, 2011). This suggests that endocannabinoid signaling in the NAc is a mechanism underlying plasticity in both neuronal structure and function. How presynaptic changes in neurotransmitter release result in post-synaptic regulation of dendritic spine structure in this system remains unclear.

Despite the importance of mGluR5 signaling in mediating the structural effects of DHT, a dose of 1 mg/kg MPEP was not able to block the conditioning effects of DHT in a CPP experiment. Although this dose of mGluR5 antagonist was sufficient to abolish NAc spine changes induced by DHT, it is possible that it is unable to block other aspects of mGluR5-mediated plasticity that may contribute to this behavior. However, it is also likely that the neurobiological mechanisms engaged by conditioning with multiple injections of DHT differ from those engaged by the single dose of DHT used in our structural experiments and may not depend on mGluR5. Indeed, across a range of drugs the role of mGluR5 in acquisition of CPP is unclear. Although some studies provide evidence that mGluR5 activity is necessary for ethanol, cocaine, morphine, and nicotine CPP (Popik and Wrobel, 2002; McGeehan and Olive, 2003; Lominac et al., 2006; Yararbas et al., 2010), others have found that blocking mGluR5 activity has no effect on CPP for amphetamine, morphine, nicotine, ethanol, or the cross sensitization of male sexual experience with amphetamine CPP (McGeehan and Olive, 2003; Pitchers et al., 2016), while others still have found that blocking mGluR5 activity may actually potentiate CPP induced by drugs of abuse (van der Kam et al., 2009; Rutten et al., 2010). Differences in the doses of MPEP and behavioral protocols used in these studies may account for their opposing results. However, like other drugs of abuse, there is a relationship between dopaminergic signaling and the conditioned effects of androgens with antagonism of both D1 and D2 dopamine receptors having been shown to abolish testosterone CPP (Schroeder and Packard, 2000). Future work may more directly study this and other molecular mechanisms of androgen reward by using intracranial self-stimulation, as the induction and expression of CPP involves a complex set of learning processes (Huston et al., 2013). Future studies may also turn to other behaviors that involve the NAcSh and are sensitive to androgen signaling, such as social and decision-making behaviors. For instance, the NAc is an important mediator of testosterone-driven intermale aggression (Albert et al., 1989, 1992), and testosterone influences behavior in a risky-decision making task that is known to depend on NAcSh activity (Stopper and Floresco, 2011; Cooper et al., 2014). Additionally, due to the close relationship of the ventral NAcSh and olfactory tubercle (Ikemoto, 2007), behavioral procedures involving olfaction may also be useful in determining the functional relevance of androgen/mGluR5 interactions.

In summary, these findings indicate a role of androgen signaling in promoting structural plasticity in a region critical in the neurocircuitry of reward. Furthermore, we provide evidence of a mechanism of androgen action involving activation of mGluR5 and endocannabinoid signaling, broadening the understanding of how androgens can exert their effects in the nervous system and supporting activation of mGluRs as a conserved mechanism across multiple steroid hormones.

## Author Contributions

KG, RM, and PM designed the research. KG and KM performed the research and analyzed the data. KG, RM, and PM wrote the paper.

## Conflict of Interest Statement

The authors declare that the research was conducted in the absence of any commercial or financial relationships that could be construed as a potential conflict of interest.
